# Global disparities in lifetime risk of lung cancer: links to socioeconomic development, smoking, and fine particulate matter from GLOBOCAN 2022

**DOI:** 10.3389/fpubh.2026.1842037

**Published:** 2026-08-12

**Authors:** Jiawei Zhai, Huijuan Zheng, Langang Xue

**Affiliations:** 1 Guangzhou Medical University, Guangzhou, Guangdong, China; 2 Department of Otolaryngology, Affiliated Hospital of Jining Medical University, Jining, Shandong, China; 3 Department of Cardiac Surgery, Second Affiliated Hospital of Fujian Medical University, Quanzhou, Fujian, China

**Keywords:** air pollution, GLOBOCAN, incidence, lifetime risk, lung cancer, mortality, smoking

## Abstract

**Background:**

Lung cancer is the leading cause of cancer-related death worldwide, with significant regional and sex differences in incidence and mortality. Although age-standardized rates are commonly used for comparisons, they do not reflect the cumulative lifetime risk, which is important for understanding the full burden of the disease because it captures the probability of an individual developing or dying from lung cancer over their lifetime.

**Methods:**

This study estimates global lifetime risk of incidence (LRI) and lifetime risk of mortality (LRM) using GLOBOCAN 2022 data and the adjusted for multiple primaries method. Socioeconomic indicators (Human Development Index, national income) came from the UN Development Programme and World Bank; air pollutant exposures from global environmental databases; and smoking-related indicators from the WHO Global Health Observatory. Associations between LRI/LRM and these factors were then explored.

**Results:**

In 2022, global LRI was 4.91%, and LRM was 4.07%. Regionally, North America exhibited the highest LRI (7.30%) and LRM (5.38%), while Africa had the lowest (LRI: 0.99%, LRM: 0.93%). Globally and across all continents, lifetime risks were significantly higher in males than in females. Regression analysis revealed that smoking prevalence was positively associated with both LRI (β = 0.001, *P* < 0.001) and LRM (β = 0.0002, *P* < 0.01). Exposure to PM_1_ was strongly linked to higher LRI (β = 0.40, *P* = 0.001) and LRM (β = 0.20, *P* = 0.006); PM_2.5_ showed similar associations (LRI: β = 0.36, *P* = 0.002; LRM: β = 0.18, *P* = 0.015). The strongest associations were observed for fine particles (PM_1_ and PM_2.5_). Furthermore, higher national socioeconomic development was positively correlated with increased LRI and LRM.

**Conclusion:**

These findings suggest that socioeconomic and environmental factors may be associated with regional disparities in lung cancer burden. This evidence could offer insights for targeted prevention policies, including smoking control and air quality management.

## Introduction

Lung cancer represents a significant global public health challenge (1, 2). Based on the most recent the Global Cancer Observatory (GLOBOCAN) 2022 projections, lung cancer ranks as the most frequently identified cancer and is the primary cause of cancer-related fatalities across the globe, with around 2.48 million new diagnoses and 1.81 million deaths reported in 2022 (3). Characterized by its high incidence and relatively low survival rates, lung cancer imposes a substantial disease burden globally (4, 5). Lung cancer contributes substantially to years of life lost (YLL) due to premature mortality and years lived with disability (YLD), reflecting its profound impact on both survival and quality of life. The burden is particularly pronounced in older adults, where both incidence and mortality peak, while a notable increase in YLL is also observed in middle-aged populations due to premature deaths (6). The epidemiological profile of the disease exhibits notable geographical and sex disparities, with the burden being particularly pronounced in Asia and among male populations (7, 8). The predominant role of smoking as a risk factor for lung cancer is supported by robust epidemiological evidence, which has extensively validated its pathogenic mechanisms at the individual level (9–11). Although the association between smoking and lung cancer has been extensively investigated, the relationship between smoking prevalence and lifetime lung cancer risk requires real-time reassessment based on the most recent, representative large-scale data, both nationally and globally, to capture current temporal and population-specific variations (12, 13).

In addition to smoking, ambient air pollution, particularly particulate matter (PM) and gaseous pollutants, has been extensively documented as a risk factor for lung cancer (14, 15). Socioeconomic factors, such as the Human Development Index (HDI) and national income, have also been linked to lung cancer burden, reflecting disparities in healthcare access, diagnostic capacity, and prevention policies, possibly due to limited healthcare resources, diagnostic delays, and inadequate preventive measures (16). Although these determinants are well-recognized, their relationship with lifetime risk indicators across diverse global populations remains insufficiently characterized.

Most current studies on the global burden of lung cancer rely on age-standardized rates (ASR) (5, 7, 17). While ASR effectively adjusts for age structure differences across populations and facilitates cross-population comparisons, it fails to intuitively reflect the cumulative incidence or mortality risk an individual faces from birth to death, which is a more direct and understandable metric for both policymakers and the general public (18). The lifetime risk of incidence (LRI) and lifetime risk of mortality (LRM) precisely provide this cumulative probability, thereby compensating for the limitation of ASR and being more suitable for policy-making and public risk communication. This advantage has been demonstrated in studies on hematologic malignancies and gastrointestinal cancer, yet the application of LRI/LRM in lung cancer research for global, cross-national comparisons remains limited (19, 20). Furthermore, existing lung cancer studies that employ lifetime risk often focus on specific countries or regions and lack a comprehensive global perspective that simultaneously assesses incidence, mortality, and their association with key risk factors like smoking and air pollution.

To address the gaps in existing research, this study introduces LRI and LRM as core evaluation indicators, estimated using the adjustment for multiple primaries (AMP) method (21, 22). This method integrates competing mortality risks, multiple primary cancer cases, and real life table data, providing a risk assessment that is more aligned with clinical realities, allowing for more equitable and comparable disease burden evaluations across countries with different population structures (2, 21, 23). The GLOBOCAN database was selected because it provides internationally comparable estimates of cancer incidence and mortality by age and sex across 185 countries, making it suitable for international comparisons and lifetime risk estimation (3). These data, together with national mortality from all causes or life table information, provide the necessary inputs for the AMP method (21). Compared with the global burden of disease (GBD), which focuses more broadly on disease burden, and the surveillance, epidemiology, and end results (SEER) program, which mainly represents the population of the United States, GLOBOCAN is more consistent with the objectives of this study. Considering this context, the aims of this research are outlined as follows: (a) to systematically evaluate the LRI and LRM for lung cancer at global, regional, and national levels, while depicting their geographical distribution and sex-based variations; (b) to investigate the connections between different indicators of smoking prevalence and the lifetime risk of lung cancer, including how these connections differ between sexes; (c) to examine the link between air pollutants and the lifetime risk of lung cancer; (d) to evaluate the relationship between the Human Development Index (HDI), economic status, and lifetime lung cancer risk, thereby providing empirical evidence to better understand the factors driving the global lung cancer burden and to aid in the development of prevention and intervention strategies.

## Materials and methods

### Data sources

Cancer incidence, mortality, and corresponding population data for 185 countries and territories were extracted from the GLOBOCAN 2022 database, which provides the most recent global cancer burden estimates (3). This resource, provided by the International Agency for Research on Cancer (IARC) through the global cancer observatory (GCO), stratifies data by sex and five-year age groups, ranging from 0–4 years to 85 years and above (3, 24). GLOBOCAN estimates are derived from the most reliable national data sources, supplemented with short-term projections and modeled mortality to incidence ratios where direct observations are unavailable. The detailed methodology has been described in prior publications and is accessible on the GCO website. The geographic classification used by GLOBOCAN groups countries into six continents and 20 world regions, including Africa, Asia, Europe, Latin America and the Caribbean, Northern America, and Oceania, with subregions such as Eastern Asia, Western Africa, and Northern Europe (3). Data for the Human Development Index (HDI) for each country were obtained from the United Nations Development Programme (UNDP) for the year 2022 (25). National income groups were categorized as high, upper-middle, lower-middle, and low income according to the World Bank historical country and lending group classifications, and were matched to 2022 gross national income (GNI) per capita using the Atlas method (26). Population-weighted annual average exposure levels (in micrograms per cubic meter, μg/m^3^) to ambient particulate matter with aerodynamic diameters equal to or less than 1 μm (PM_1_), 2.5 μm (PM_2.5_), 10 μm (PM_10_), and nitrogen dioxide (NO_2_) were sourced from the State of Global Air 2022 report (27). Sulfur dioxide (SO__2__) and ozone (O_3_) data for the same year were obtained from the Emissions Database for Global Atmospheric Research (EDGAR) (28) and Open Weather (29), respectively. Data regarding smoking prevalence were sourced from the Global Health Observatory repository of the World Health Organization. Two primary metrics were used: (1) the prevalence of current cigarette smoking among persons aged 15 years and older, and (2) the prevalence of current tobacco use (including both smoked and smokeless tobacco products) among persons aged 15 years and older (30).

### Calculation of lifetime risk

The LRI and LRM of lung cancer were derived using the adjusted for multiple primaries (AMP) method, which accounts for competing causes of death and the occurrence of multiple primary cancers (2, 21). The core formulas are as follows.


$$\begin{array}{l} For\;LRI:\;LRI\;=\;\Sigma_{i}[(R_{i}/\;(R_{i}+\;M_{i}\;D_{i}))\;\times {\hat{S}}_{0}(a_{i})\\ \times \;(1-exp(-w_{i}/N_{i}\times \;(R_{i}+\;M_{i}-D_{i})))]\\ For\;LRM:\;LRM\;=\;\Sigma_{i}[(R_{i}^{\prime }/\;(R_{i}^{\prime }+\;M_{i}-D_{i}))\;\times {\hat{S}}_{0}(a_{i})\\ \times \;(1-exp(-w_{i}/N_{i}\times \;(R_{i}^{\prime }+\;M_{i}-D)))] \end{array}$$

where for each age group i:

*R* = number of incident lung cancer cases (for LRI);
*R*′ = number of lung cancer deaths (for LRM);
*M* = all-cause deaths;
*D* = cancer deaths (all types, used to avoid double-counting);
*N* = population size;
*w* = width of the age interval (5 years for most groups, with the open-ended 85+ interval set to 1);

Ŝ*0*(*a*_*i*_) = probability of being alive and cancer-free at the start of age interval *i*, estimated from life tables (see Supplementary Materials for recursive calculation).

This approach estimates the cumulative probability that an individual will develop or die from cancer during their remaining lifespan by integrating age-specific rates with current life table data. Detailed derivations, including variance calculations for confidence intervals, are provided in the Supplementary Materials.

### Statistical analysis

All analyses were stratified by sex (male, female, and both sexes combined) and by geographic scale, which included individual countries, continents, United Nations regions, and the global aggregate. To enable socioeconomic comparisons, results were further categorized based on HDI levels and World Bank income classifications.

We examined the association between tobacco exposure and lung cancer risk by analyzing national estimates of tobacco use, smoking prevalence, and cigarette consumption in relation to the corresponding LRI and LRM. The associations of LRI and LRM with HDI, income levels, and tobacco indicators were first visualized using scatter plots overlaid with linear regression lines. Linear regression analyses were then performed to evaluate the relationship between smoking prevalence and both LRI and LRM. Sex-stratified linear regression models were employed to examine potential effect modification by sex, with separate models fitted for males and females.

The association between ambient air pollution and lung cancer risk was assessed using multivariable linear regression. Separate models were fitted for each pollutant. A total of six air pollutants (PM_1_, PM_2.5_, PM_10_, NO_2_, SO_2_, and O_3_) were examined. Effect sizes (β coefficients), representing the absolute change in lifetime risk (in percentage points) per 1 ug/m^3^ increase in pollutant concentration, and their 95% CIs were reported for each pollutant.

The Harvey–Collier test was used to assess the linearity assumption of the regression model. This test is based on recursive residuals, with the null hypothesis of correct model specification (*P* > 0.05 supports linearity). The test was applied to LRI and LRM outcomes, stratified by sex.

To investigate the potential interaction between smoking and air pollution on lifetime lung cancer risk, we included multiplicative interaction terms between three smoking indicators (smoking prevalence, cigarette smoking prevalence, and tobacco use prevalence) and six air pollutants (PM_1_, PM_2.5_, PM_10_, NO_2_, and O_3_) in multivariable linear regression models. Interaction results were presented as coefficients with 95% CIs and *P*-values.

Sensitivity analyses were performed by comparing the full lifetime risk estimates (LRI and LRM) with the cumulative risk to age 74, aimed at evaluating the robustness of the findings to the inclusion of older-age data. All data visualizations and statistical analyses were carried out utilizing R software (version 4.3.3).

## Results

### Geographic disparities in the lifetime risk of lung cancer

**Figure 1:**
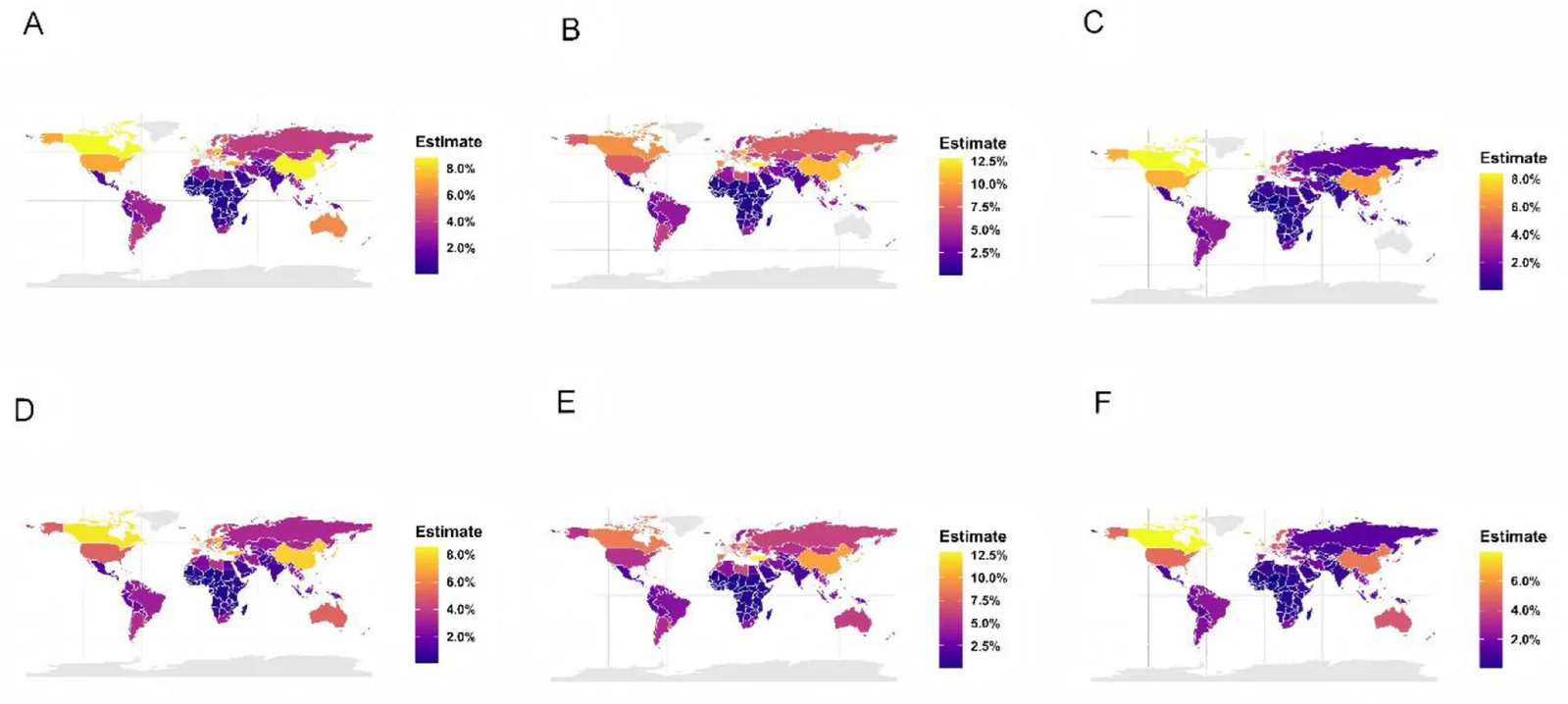
Global lifetime risk (%) of lung cancer incidence (LRI) and mortality (LRM), 2022. **(A)** LRI: both sexes; **(B)** LRI: males; **(C)** LRI: females; **(D)** LRM: both sexes; **(E)** LRM: males; **(F)** LRM: females.

The AMP method accounts for age structure. Age differences are incorporated into the lifetime risk estimates. In 2022, the global LRI and LRM from lung cancer were estimated to be 4.91% (95% CI: 4.91–4.92) and 4.07% (95% CI: 4.07–4.08), respectively. Significant geographical variations in both LRI and LRM were observed across the continents. North America exhibited the highest LRI (7.30%; 95% CI: 7.28–7.32), followed by Europe (5.90%; 95% CI: 5.89–5.91), Oceania (5.75%; 95% CI: 5.68–5.81), and Asia (5.34%; 95% CI: 5.33–5.34). In contrast, Latin America and the Caribbean demonstrated a much lower LRI (2.75%; 95% CI: 2.74–2.76), while Africa had the lowest value (0.99%; 95% CI: 0.99–1.00). A similar pattern was observed for LRM, with North America exhibiting the highest risk (5.38%; 95% CI: 5.36–5.40), followed by Europe (5.10%; 95% CI: 5.09–5.12), Oceania (4.64%; 95% CI: 4.58–4.70), and Asia (4.51%; 95% CI: 4.50–4.51), and Africa showing the lowest LRM (0.93%; 95% CI: 0.92–0.94). Both LRI and LRM were significantly higher in males than in females globally, with this sex-based disparity being consistently observed across all continents. These findings highlight substantial geographic and sex-related disparities in lung cancer risk, with higher burdens in more economically developed regions, such as North America and Europe, compared to regions like Africa, which experience a considerably lower disease burden (Figure 1, Supplementary Table S1).

**Figure 2:**
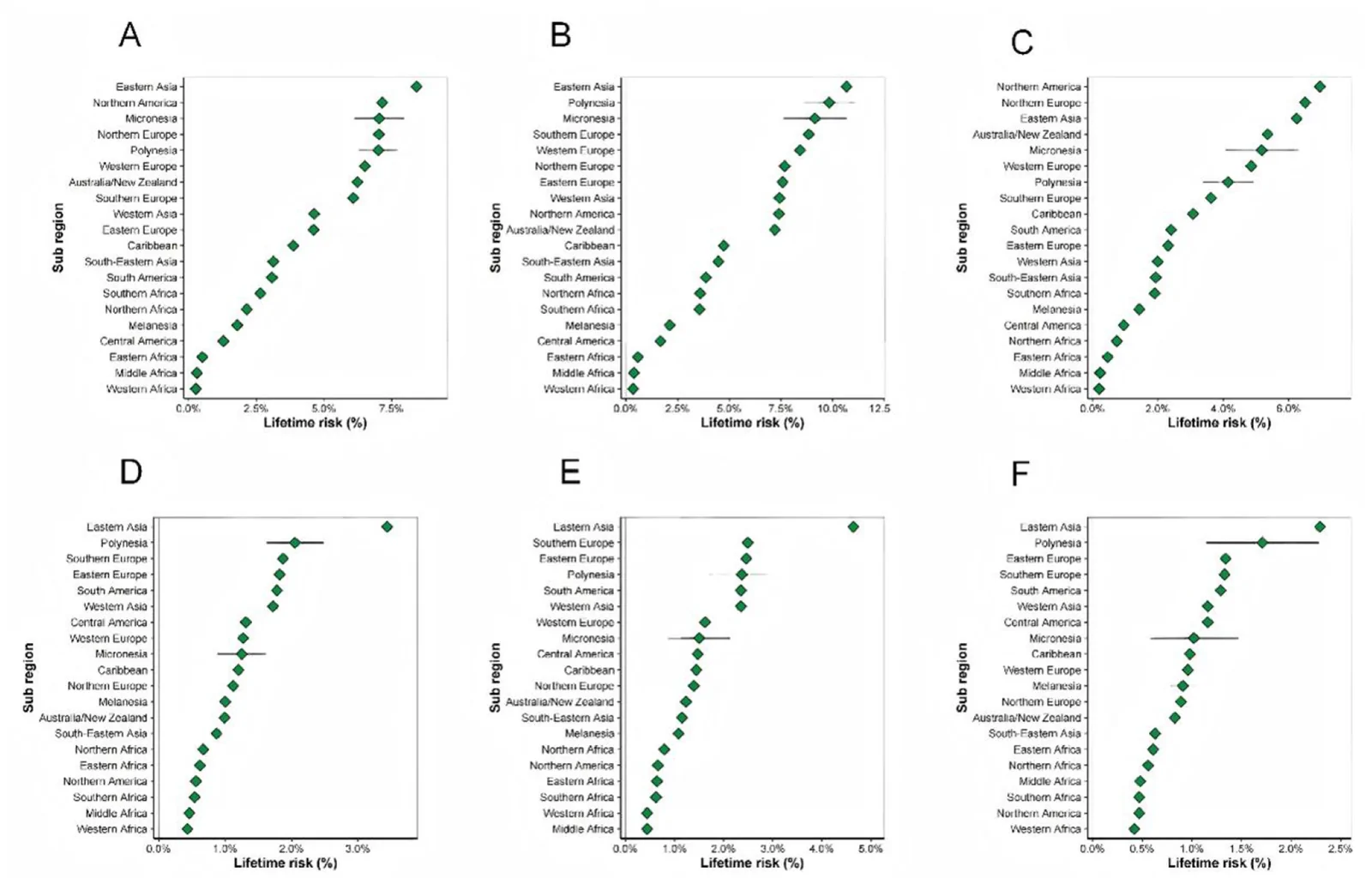
Lifetime risk (%) of lung cancer incidence (LRI) and mortality (LRM) by region, 2022. **(A)** LRI: both sexes; **(B)** LRI: males; **(C)** LRI: females; **(D)** LRM: both sexes; **(E)** LRM: males; **(F)** LRM: females.

Regional analysis of LRI revealed marked variability across different regions. Eastern Asia reported the highest LRI (8.60%, 95% CI: 8.58%−8.61%), while Western Africa exhibited the lowest (0.27%, 95% CI: 0.26%−0.28%). In economically developed regions, elevated LRI rates were observed in Eastern Asia (8.60%), Northern America (7.30%), Northern Europe (7.20%), Western Europe (6.66%), and Australia/New Zealand (6.37%). In contrast, most other regions, including Eastern Europe, Southern Europe (6.20%), Western Asia, South-Eastern Asia, South America, Central America, the Caribbean, Melanesia, Micronesia, and Polynesia, recorded LRI values below 5.00%, with the exception of Southern Europe (6.20%). Notably, all African subregions (Eastern, Central, Northern, Western, and Southern Africa) consistently demonstrated low LRI levels. A similar geographical pattern was evident for LRM, with Eastern Asia showing the highest LRM (7.50%, 95% CI: 7.48%−7.51%), while Western Africa had the lowest (0.26%, 95% CI: 0.26%−0.27%). The distribution of LRM across regions mirrored that of LRI, with higher estimates observed in high-income regions such as Eastern Asia, Northern America (5.38%), Northern Europe (6.14%), Western Europe (5.95%), and Australia/New Zealand (5.29%). Conversely, lower rates of LRM were found in Africa and parts of Asia and Oceania. At the global level, both LRI and LRM were significantly higher in males than in females, and this sex-based disparity was evident across all regions (Figure 2, Supplementary Table S2).

### Smoking shows a significant association with the lifetime risk of lung cancer

**Figure 3:**
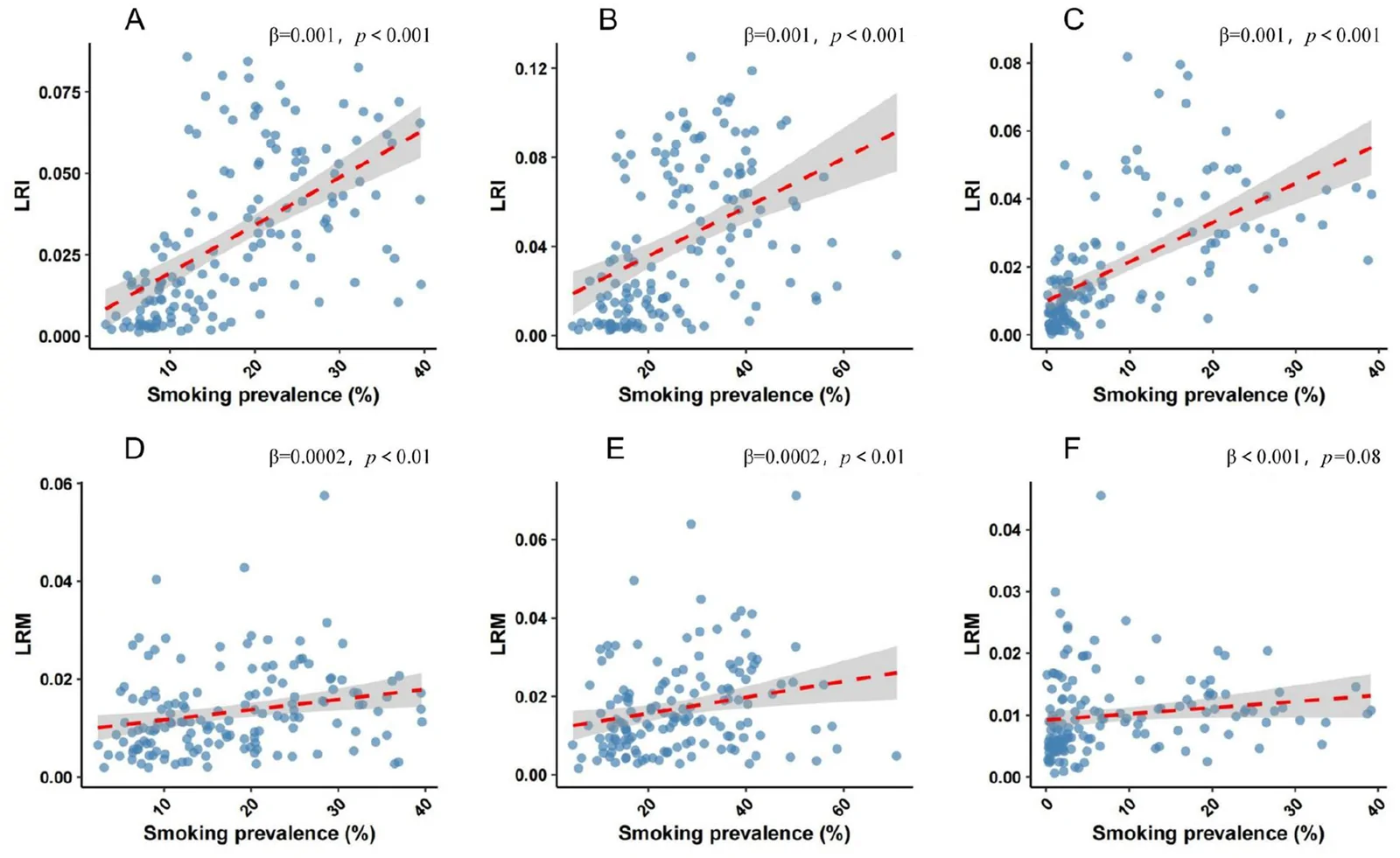
The association between smoking prevalence and lifetime lung cancer risk in 2022. **(A)** LRI: both sexes; **(B)** LRI: males; **(C)** LRI: females; **(D)** LRM: both sexes; **(E)** LRM: males; **(F)** LRM: females.

**Figure 4:**
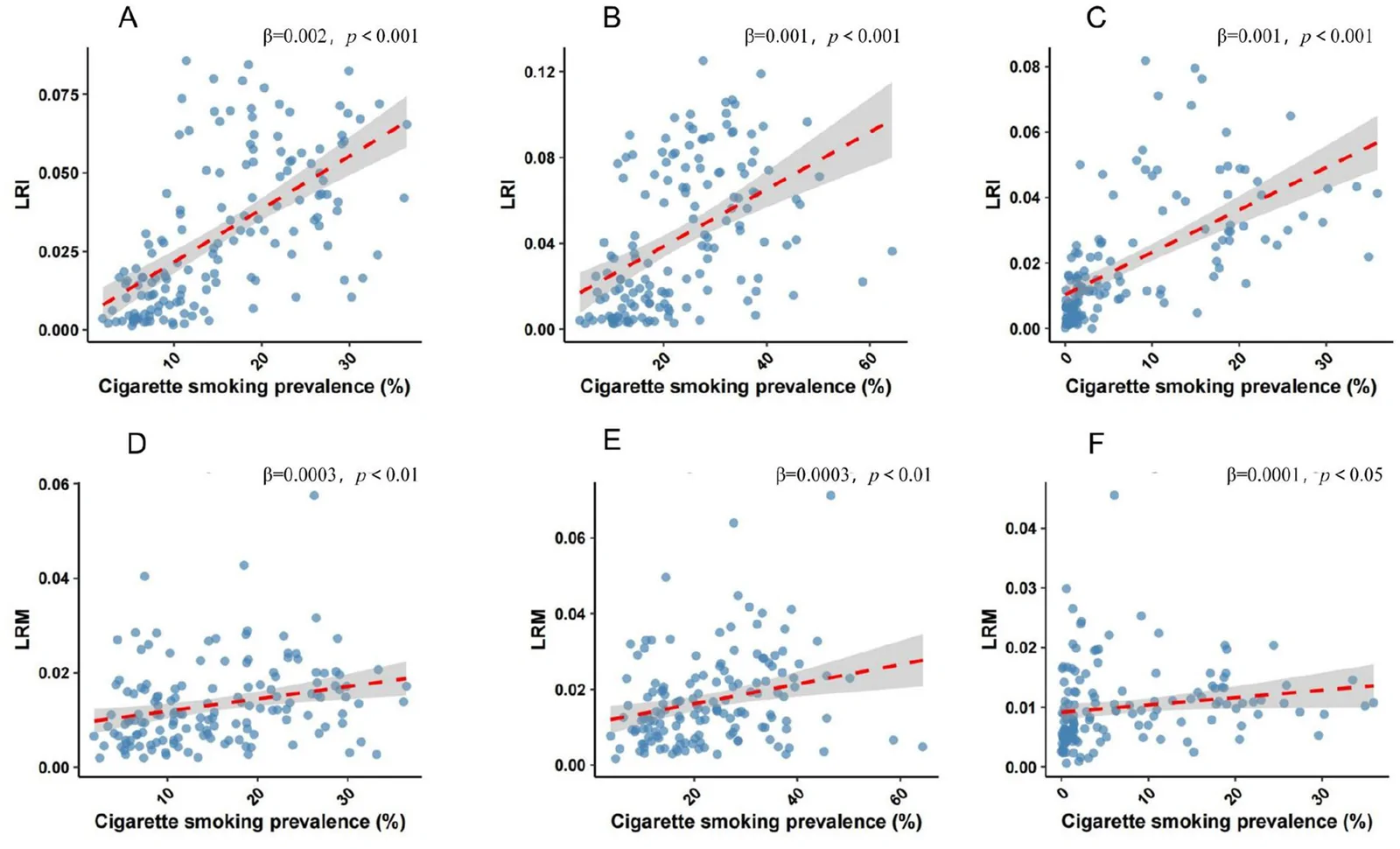
The association between cigarette smoking prevalence and lifetime lung cancer risk in 2022. **(A)** LRI: both sexes; **(B)** LRI: males; **(C)** LRI: females; **(D)** LRM: both sexes; **(E)** LRM: males; **(F)** LRM: females.

**Figure 5:**
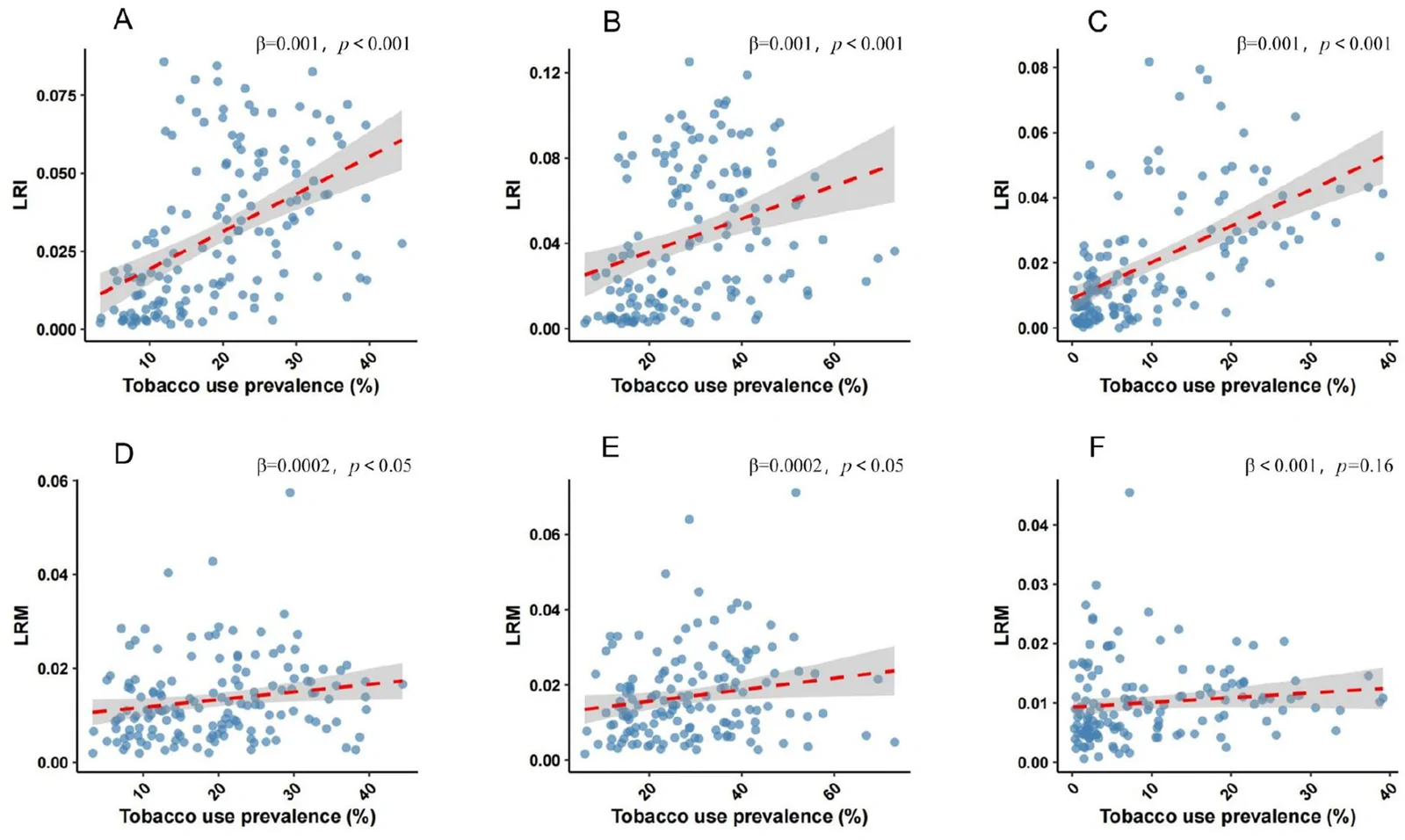
The association between tobacco use prevalence and lifetime lung cancer risk in 2022. **(A)** LRI: both sexes; **(B)** LRI: males; **(C)** LRI: females; **(D)** LRM: both sexes; **(E)** LRM: males; **(F)** LRM: females.

In the full population analysis, smoking prevalence was significantly associated with both LRI (β = 0.001, *P* < 0.001) and LRM (β = 0.0002, *P* < 0.01). Sex-stratified analyses revealed that while LRI remained significantly associated with smoking in both males and females, a significant association with LRM was observed only in males (β = 0.0002, *P* < 0.01). In contrast, the association between smoking and LRM in females was not statistically significant. These findings highlight a clear sex-based disparity in the impact of smoking on lung cancer mortality (Figure 3, Supplementary Table S4). A significant positive association was observed between cigarette smoking prevalence and both lung cancer incidence (LRI: β = 0.002, *P* < 0.001) and lung cancer mortality (LRM: β = 0.0003, *P* < 0.01) in the overall population. Although this association was consistent in both sexes, it was markedly stronger in males, as indicated by higher regression coefficients, suggesting a greater susceptibility to smoking-related lung cancer in men (Figure 4, Supplementary Table S5). Significant associations were also observed between tobacco use prevalence and both LRI (β = 0.001, *P* < 0.001) and LRM (β = 0.0002, *P* < 0.05) in the overall population. However, sex-specific analyses revealed a notable disparity: the associations were significant for both outcomes in males, but only for LRI in females, further indicating that the relationship between smoking and LRM differs by sex (Figure 5, Supplementary Table S6). Notably, the prevalence of all three smoking indicators (current smoking, current cigarette smoking, and current tobacco use) was consistently higher in males than in females (Supplementary Figure S1). The Harvey-Collier test confirmed that all main effect regression models satisfied the linearity assumption (all *P* > 0.05) (Supplementary Table S7).

### Exposure to airborne fine particulate matter is associated with increased lifetime risk of lung cancer

**Figure 6:**
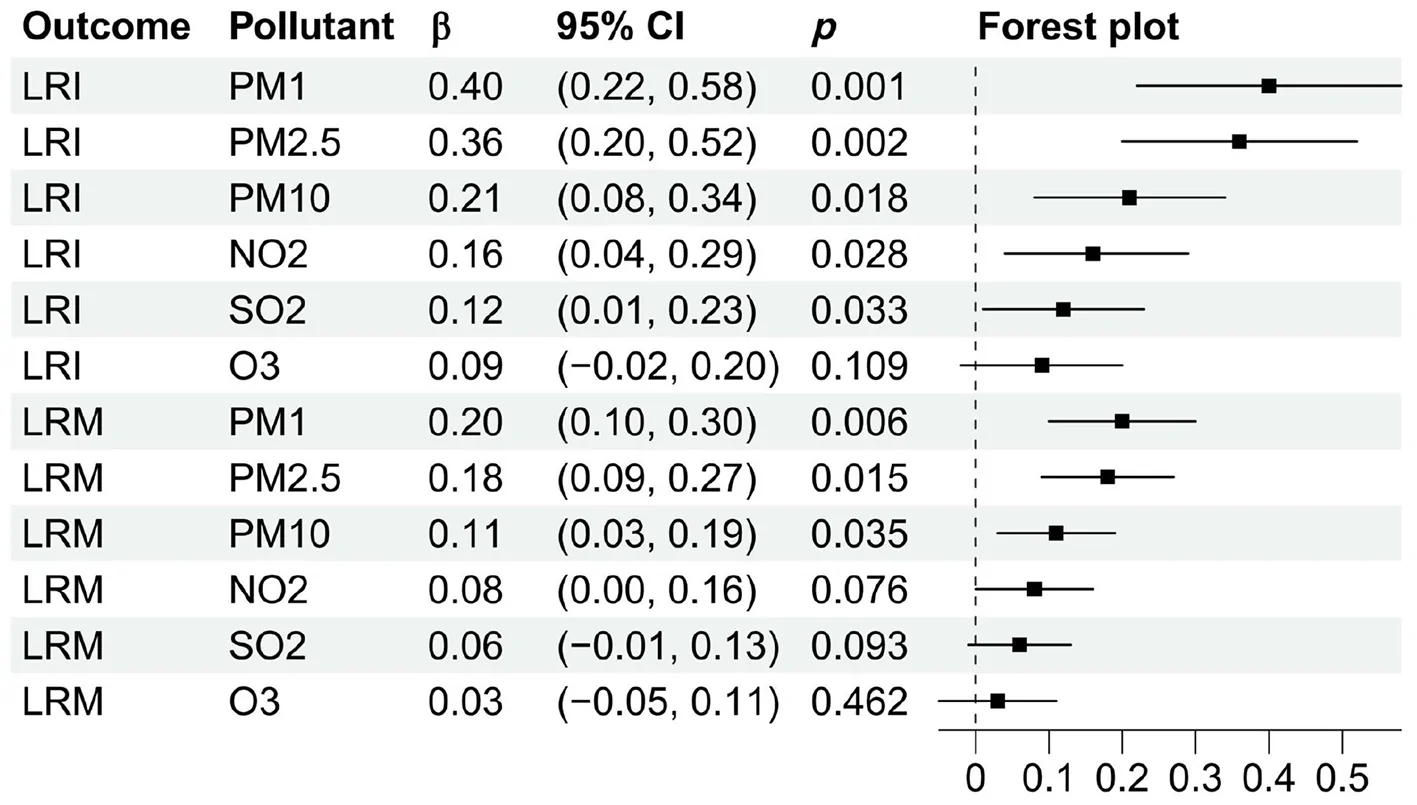
Air pollution and lifetime risk of developing or dying from lung cancer, 2022.

Significant positive correlations were observed between particulate matter (PM) pollutants and both the LRI and LRM from lung cancer, with the strength of these associations varying according to particle size. For LRI, all particulate pollutants were significantly associated with an increased risk. The strongest association was observed for PM_1_ (β = 0.40, 95% CI: 0.22–0.58, *P* = 0.001), followed by PM_2.5_ (β = 0.36, 95% CI: 0.20–0.52, *P* = 0.002) and PM_10_ (β = 0.21, 95% CI: 0.08–0.34, *P* = 0.018). These results suggest a trend of increasing risk with decreasing particle size. The effect estimate for NO_2_ was comparatively modest (β = 0.16, 95% CI: 0.04–0.29, *P* = 0.028), yet the association with LRI remained statistically significant. In addition, SO_2_ was also significantly associated with LRI (β = 0.12, 95% CI: 0.01–0.23, *P* = 0.033), while O_3_ was not (*P* = 0.109). With respect to LRM, significant positive correlations were also observed for PM_1_ (β = 0.20, 95% CI: 0.10–0.30, *P* = 0.006), PM_2.5_ (β = 0.18, 95% CI: 0.09–0.27, *P* = 0.015), and PM_10_ (β = 0.11, 95% CI: 0.03–0.19, *P* = 0.035), showing a consistent pattern with the findings for LRI. In contrast, the associations between LRM and NO_2_, SO_2_, and O_3_ were not statistically significant (Figure 6). To assess whether the associations differ by sex, sex-stratified analyses were performed (Supplementary Figures 2, 3 which showed highly similar effect estimates between males and females for all six pollutants, with no clear sex difference observed. No significant interactions were detected between any of the three smoking indicators and the six air pollutants (all *P* > 0.05), indicating that the associations between air pollution and lung cancer risk were not significantly modified by smoking (Supplementary Figure S4).

### Positive association between national socioeconomic development and lifetime risk of lung cancer

When the HDI was modeled as a continuous variable, it showed a significant positive association with both LRI and LRM from lung cancer. In the overall population, each unit increase in HDI was associated with a rise in LRM (β = 0.13, *P* < 0.001) and LRI (β = 0.17, *P* < 0.001). This positive correlation was consistent in both sexes and was visually reinforced by a gradient where regions with higher life expectancy, indicative of higher HDI, corresponded to greater lifetime risks (Supplementary Figure S5 and Supplementary Table S3). To further explore this socioeconomic gradient, we categorized countries by HDI and World Bank income levels. Both analyses revealed significant trends (*P* < 0.001). Countries with higher HDI or income levels demonstrated progressively greater LRI and LRM (Supplementary Figures S6, S7). These findings indicate a positive association between socioeconomic development and lung cancer burden, which was consistent for both males and females.

### Sensitivity analysis

Sensitivity analysis revealed a strong linear relationship between the lifetime risk estimates and the cumulative risks derived from GLOBOCAN 2022 for individuals aged 0–74 years. This was evident from the tight clustering of data points along the line of equality (*y* = x) in the scatter plot, indicating a high degree of concordance between the two risk estimates. The observed pattern was consistent across both the overall population and sex-specific subgroups. These findings collectively support the robustness and reliability of the methodology employed in this study (Supplementary Figure S8).

## Discussion

Utilizing the GLOBOCAN 2022 data, this research conducted a systematic assessment of the LRI and LRM from lung cancer on global, regional, and national scales. The findings reveal that the global rates for LRI and LRM were 4.91 and 4.07%, correspondingly. The data presented are considerably greater than those related to the majority of other cancer categories, highlighting that lung cancer continues to be a significant factor in the worldwide cancer burden (24, 31). Notable disparities were observed by sex, with males generally exhibiting higher lifetime risks of lung cancer than females. Marked geographical variations were also evident, with notable differences observed across regions, including Eastern Asia, Eastern Europe, and Central America, reflecting the complex patterns of lung cancer burden worldwide (32–34). Beyond these regions, the GBD framework classifies North Africa and the Middle East, often referred to as the MENA region, as a distinct geographic unit; GBD-based analyses indicate that this region faces a substantial smoking-related disease burden, with rising trends observed in several countries (35, 36).

Additionally, we observed a positive ecological association between national socioeconomic development, as measured by HDI and income level, and the lifetime risk of lung cancer incidence and mortality. As shown in Supplementary Supplementary Figures S5–S7, higher HDI and income levels were generally associated with higher LRI and LRM. In categorical analyses, LRI and LRM increased across HDI and World Bank income groups, and HDI as a continuous variable was also positively associated with both outcomes. This pattern reflects the construction of lifetime risk, which depends not only on age-specific lung cancer risk but also on population survival structure. The AMP method estimates lifetime risk by integrating age-specific lung cancer incidence or mortality with all-cause survival and competing mortality across the lifespan (37). Therefore, higher LRI and LRM in countries with higher HDI or income may partly reflect longer life expectancy and lower competing mortality, which increase the probability of surviving to older ages when lung cancer is more common. This is consistent with life expectancy being a component of HDI (38). In addition, more complete cancer registration and greater diagnostic ascertainment in higher-HDI settings may contribute to higher observed lifetime risk estimates (39). Taken together, these findings suggest that the observed association between socioeconomic development and lifetime lung cancer risk should be interpreted in the context of demographic structure, survival patterns, and data quality. Of note, as an ecological, country-level analysis, these results do not contradict individual-level evidence on socioeconomic deprivation and cancer risk, as they reflect different levels of inference.

A systematic examination was conducted of the relationships between three indicators of smoking prevalence (cigarette smoking, smoking, and tobacco use) and the lifetime risk of lung cancer. The results revealed that all three indicators were significantly positively associated with both LRI and LRM of lung cancer, with notable sex heterogeneity observed. Among males, all indicators demonstrated stronger effect estimates and greater statistical significance, particularly for lifetime risk mortality. In females, although a positive trend was observed, the associations were mostly not statistically significant, and the effect estimates were substantially lower than those in males. These findings are consistent with the report by Thun et al. on the cohort of people who currently smoke in the United States, supporting the view that males may be more susceptible to tobacco carcinogens (40). Potential mechanisms include differences in hormonal metabolism, smoking behavior patterns such as deep inhalation and quantity of cigarettes smoked, and synergistic effects of occupational exposures (41, 42). Furthermore, the weaker association observed among women may partly reflect attenuation of the risk signal due to the use of aggregate national-level exposure data (43). Specifically, national average smoking prevalence may obscure substantial heterogeneity in smoking behaviors among women. In populations where most women do not smoke, the risk associated with a smaller subgroup of women with heavier smoking exposure may be attenuated when smoking exposure is represented only by national-level average prevalence (44, 45). According to Pirie et al. in the Million Women Study conducted in the United Kingdom, the overall cumulative exposure to tobacco for individuals might act as a more accurate indicator of risk (46). Finally, although our study focuses on the overall population, a recent meta-analysis suggests that never-smoking women may have an elevated risk of lung cancer (47), indicating that factors beyond smoking warrant further investigation.

In addition to smoking, this study explored the potential associations of six common air pollutants, namely PM_1_, PM_2.5_, PM_10_, NO_2_, SO_2_ and O_3_, with the lifetime risk of lung cancer. The analysis revealed positive correlations between particulate matter pollutants and both LRI and LRM. Notably, smaller particles generally showed stronger associations. Specifically, fine particulate matters (PM_1_ and PM_2.5_) demonstrated higher association strengths than the larger-sized PM_10_ and the gaseous pollutants. This pattern aligns with the wider literature, where NO__2__ is often considered a marker of traffic-related pollution rather than a direct carcinogen (15). Using global data, this study provides further evidence supporting the association between fine particulate matter exposure and the lifetime risk of lung cancer incidence and mortality. Of note, these associations have also been observed among never-smokers in prior studies, suggesting that particulate matter may act as an independent risk factor beyond tobacco exposure (48, 49). These findings are consistent with previous studies that reported positive associations between fine particulate matter exposure and lung cancer outcomes. For instance, Guo et al. identified a positive relationship between PM_1_ exposure and lung cancer incidence among Chinese women (50), while Wu et al. and Hung et al. demonstrated similar associations between PM_2.5_ exposure and lung cancer mortality (51, 52). Additionally, recent investigations by Bouma et al. and Pond et al. have linked ultrafine particulate matter exposure to lung cancer deaths (53, 54). Together with these prior reports, our findings underscore the potential health threat posed by fine and ultrafine particulate matter and highlight the importance of addressing such pollution in lung cancer prevention and control strategies.

A key methodological strength of this research is its systematic evaluation of LRI and LRM using the recently published GLOBOCAN 2022 database with global, regional, and national coverage. The use of the AMP method accounts for competing mortality risks and age structure. The multi-factorial analytical framework examines associations with socioeconomic development, smoking prevalence, and air pollution. However, several limitations should be acknowledged. First, cancer registry quality varies across countries, and underreporting in lower-HDI regions may lead to underestimation of LRI and LRM. Second, the smoking metrics are population-level prevalence rates that cannot capture individual cumulative exposure or historical trends, and GLOBOCAN 2022 data are not stratified by smoking status. Third, without region-stratified analyses, we cannot provide region-specific estimates or quantify how much smoking and air pollution explain regional disparities.

## Conclusion

In summary, this study suggests that lifetime risk indicators (LRI and LRM) may offer a more comprehensive assessment of the true burden of lung cancer at the population level. According to our research, we suggest that, alongside persistent tobacco control initiatives, strategies for managing air quality that focus on fine particulate matter, including PM_2.5_ and PM_1_, should be incorporated into the national cancer control framework as a vital approach to lowering the lifetime risk of lung cancer among the broader population.

## Data Availability

Publicly available datasets were analyzed in this study. This data can be found here: The data supporting this study are publicly accessible and were obtained from the GLOBOCAN 2022 database, available online at https://gco.iarc.fr/.
