# Supplementary material for: Global disparities in lifetime risk of lung cancer: links to socioeconomic development, smoking, and fine particulate matter from GLOBOCAN 2022
Source: Front Public Health. 2026 Aug 12;14:1842037. doi: 10.3389/fpubh.2026.1842037 (PMC13506773; doi:10.3389/fpubh.2026.1842037)
Supplement: Supplementary file 1 [file Table_1.docx]

**Supplementary Information (SI)**

**Global Disparities in Lifetime Risk of Lung Cancer: Links to Socioeconomic Development, Smoking, and Fine Particulate Matter from GLOBOCAN 2022**

**A. Supplementary tables**

**Tables S1 to S7**

**B.** **Supplementary figures**

**Figures S1 to S8**

**C. Methods for calculating lifetime risk of developing or dying from cancer**

**.**

A.

Table S1. Lifetime risks (%) of lung cancer incidence and mortality in 2022 by five continents.

| Region.name | LRI (%) (95% CI) | LRM (%) (95% CI) |
| --- | --- | --- |
| Africa | 0.99% (0.99%–1.00%) | 0.93% (0.92%–0.94%) |
| Latin America and the Caribbean | 2.75% (2.74%–2.76%) | 2.47% (2.46%–2.48%) |
| Northern America | 7.30% (7.28%–7.32%) | 5.38% (5.36%–5.40%) |
| Europe | 5.90% (5.89%–5.91%) | 5.10% (5.09%–5.12%) |
| Oceania | 5.75% (5.68%–5.81%) | 4.64% (4.58%–4.70%) |
| Asia | 5.34% (5.33%–5.34%) | 4.51% (4.50%–4.51%) |
| Total | 4.91% (4.91%–4.92%) | 4.07% (4.07%–4.08%) |

LRI：lifetime risk of lung cancer incidence, LRM：lifetime risk of lung cancer mortality.

Table S2. Lifetime risks (%) of lung cancer incidence and mortality in 2022 by region.

| Region.name | LRI (%) (95% CI) | LRM (%) (95% CI) |
| --- | --- | --- |
| Africa |  |  |
| Eastern Africa | 0.51% (0.50%-0.52%) | 0.49% (0.48%-0.50%) |
| Middle Africa | 0.31% (0.30%-0.32%) | 0.30% (0.29%-0.31%) |
| Northern Africa | 2.17% (2.14%-2.19%) | 2.00% (1.98%-2.02%) |
| Southern Africa | 2.68% (2.64%-2.73%) | 2.57% (2.52%-2.62%) |
| Western Africa | 0.27% (0.26%-0.28%) | 0.26% (0.26%-0.27%) |
| Asia |  |  |
| Eastern Asia | 8.60% (8.58%-8.61%) | 7.50% (7.48%-7.51%) |
| South-Eastern Asia | 3.17% (3.16%-3.19%) | 2.95% (2.93%-2.96%) |
| Western Asia | 4.73% (4.70%-4.76%) | 4.65% (4.62%-4.69%) |
| Europe |  |  |
| Eastern Europe | 4.69% (4.67%-4.70%) | 4.04% (4.02%-4.06%) |
| Northern Europe | 7.20% (7.16%-7.24%) | 6.14% (6.10%-6.18%) |
| Southern Europe | 6.20% (6.17%-6.23%) | 5.67% (5.64%-5.69%) |
| Western Europe | 6.66% (6.63%-6.69%) | 5.95% (5.92%-5.98%) |
| Americas |  |  |
| Caribbean | 3.93% (3.87%-3.98%) | 3.41% (3.36%-3.46%) |
| Central America | 1.29% (1.27%-1.31%) | 1.22% (1.20%-1.24%) |
| Northern America | 7.30% (7.28%-7.32%) | 5.38% (5.36%-5.40%) |
| South America | 3.10% (3.08%-3.11%) | 2.78% (2.76%-2.80%) |
| Oceania |  |  |
| Australia/New Zealand | 6.37% (6.29%-6.45%) | 5.29% (5.22%-5.36%) |
| Melanesia | 1.81% (1.71%-1.92%) | 1.62% (1.52%-1.72%) |
| Micronesia | 7.33% (6.35%-8.30%) | 7.24% (6.27%-8.22%) |
| Polynesia | 7.20% (6.46%-7.95%) | 6.80% (6.06%-7.54%) |

LRI：lifetime risk of lung cancer incidence, LRM：lifetime risk of lung cancer mortality.

Table S3. Lifetime risks (%) of lung cancer incidence and mortality by HDI in 2022.

| Exposure Variable | Sex | LRI (β) | LRI (*P* value) | LRM (β) | LRM (P value) |
| --- | --- | --- | --- | --- | --- |
| HDI | Both sexes | 0.15 | ＜0.001 | 0.08 | ＜0.001 |
| HDI | Male | 0.11 | ＜0.001 | 0.17 | ＜0.001 |
| HDI | LRI Female | 0.09 | ＜0.001 | 0.13 | ＜0.001 |

HDI：Human Development Index, LRI：lifetime risk of lung cancer incidence, LRM：lifetime risk of lung cancer mortality.

Table S4. The association between smoking prevalence and the lifetime risk of lung cancer incidence and mortality in 2022.

| Exposure Variable | Sex | LRI (β) | LRI (*P* value) | LRM (β) | LRM (*P* value) |
| --- | --- | --- | --- | --- | --- |
| Smoking prevalence | Both sexes | 0.001 | ＜0.001 | 0.0002 | ＜0.01 |
| Smoking prevalence | Male | 0.001 | ＜0.001 | 0.0002 | ＜0.01 |
| Smoking prevalence | Female | 0.001 | ＜0.001 | ＜0.001 | 0.08 |

LRI：lifetime risk of lung cancer incidence, LRM：lifetime risk of lung cancer mortality.

Table S5. The association between cigarette smoking prevalence and the lifetime risk of lung cancer incidence and mortality in 2022.

| Exposure Variable | Sex | LRI (β) | LRI (*P* value) | LRM (β) | LRM (*P* value) |
| --- | --- | --- | --- | --- | --- |
| cigarette smoking prevalence | Both sexes | 0.002 | ＜0.001 | 0.0003 | ＜0.01 |
| cigarette smoking prevalence | Male | 0.001 | ＜0.001 | 0.0003 | ＜0.01 |
| cigarette smoking prevalence | Female | 0.001 | ＜0.001 | 0.0001 | ＜0.05 |

LRI：lifetime risk of lung cancer incidence, LRM：lifetime risk of lung cancer mortality.

Table S6. The association between tobacco use prevalence and the lifetime risk of lung cancer incidence and mortality in 2022.

| Exposure Variable | Sex | LRI (β) | LRI (*P* value) | LRM (β) | LRM (*P* value) |
| --- | --- | --- | --- | --- | --- |
| Tobacco use prevalence | Both sexes | 0.001 | ＜0.001 | 0.0002 | ＜0.05 |
| Tobacco use prevalence | Male | 0.001 | ＜0.001 | 0.0002 | ＜0.05 |
| Tobacco use prevalence | Female | 0.001 | ＜0.001 | ＜0.001 | 0.16 |

LRI：lifetime risk of lung cancer incidence, LRM：lifetime risk of lung cancer mortality.

Table S7. Harvey-Collier test for linearity assumption of regression models predicting lung cancer lifetime risk by sex.

| Indicator Code | Sex | LRI(β) | LRI (*P* value) | Harvey-Collier *P* | LRM (β) | LRM（*P* value) | Harvey-Collier  *P* |
| --- | --- | --- | --- | --- | --- | --- | --- |
| tobacco use prevalence | Both sexes | 0.007 | <0.001 | 0.428 | 0.011 | 0.029 | 0.185 |
| smoking prevalence | Both sexes | 0.005 | <0.001 | 0.651 | 0.010 | 0.005 | 0.349 |
| cigarette smoking prevalence | Both sexes | 0.005 | <0.001 | 0.712 | 0.009 | 0.001 | 0.522 |
| tobacco use prevalence | Male | 0.020 | <0.001 | 0.476 | 0.013 | 0.035 | 0.882 |
| smoking prevalence | Male | 0.014 | <0.001 | 0.298 | 0.012 | 0.007 | 0.154 |
| cigarette smoking prevalence | Male | 0.013 | <0.001 | 0.614 | 0.011 | 0.002 | 0.337 |
| tobacco use prevalence | Female | 0.009 | <0.001 | 0.552 | 0.009 | 0.163 | 0.725 |
| smoking prevalence | Female | 0.010 | <0.001 | 0.419 | 0.009 | 0.076 | 0.244 |
| cigarette smoking prevalence | Female | 0.010 | <0.001 | 0.683 | 0.009 | 0.046 | 0.191 |

LRI：lifetime risk of lung cancer incidence, LRM：lifetime risk of lung cancer mortality.

B.

**Figure S1. Sex differences in prevalence of three smoking indicators.**

**Figure S2. Associations between air pollutants and lifetime lung cancer risk in females, 2022**

**Figure S3. Associations between air pollutants and lifetime lung cancer risk in males, 2022.**

**Figure S4. Forest plot of interaction effects between smoking indicators and air pollutants.**

**Figure S5. Lifetime risks (%) of lung cancer incidence and mortality by HDI in 2022.**

（A）LRI: Both sexes; (B) LRI: Males; (C) LRI: Females; (D) LRM: Both sexes; (E) LRM: Males; (F) LRM: Females. LRI：lifetime risk of lung cancer incidence, LRM：lifetime risk of lung cancer mortality.

**Figure S6. Lifetime risks (%) of lung cancer incidence and mortality by HDI categories in 2022.**

（A）LRI: Both sexes; (B) LRI: Males; (C) LRI: Females; (D) LRM: Both sexes; (E) LRM: Males; (F) LRM: Females. LRI：lifetime risk of lung cancer incidence, LRM：lifetime risk of lung cancer mortality.

**Figure S7. Lifetime risks (%) of lung cancer incidence and mortality by National income categories in 2022.**

（A）LRI: Both sexes; (B) LRI: Males; (C) LRI: Females; (D) LRM: Both sexes; (E) LRM: Males; (F) LRM: Females. LRI：lifetime risk of lung cancer incidence, LRM：lifetime risk of lung cancer mortality.

**Figure S8. Comparisons of the lifetime risks and the cumulative risks for 0-74 age groups of LRI and LRM from lung cancer.**

（A）LRI: Both sexes; (B) LRI: Males; (C) LRI: Females; (D) LRM: Both sexes; (E) LRM: Males; (F) LRM: Females. LRI：lifetime risk of lung cancer incidence, LRM：lifetime risk of lung cancer mortality.

**C. Methods for calculating lifetime risk of developing or dying from cancer**

The lifetime risk of developing cancer mentioned in this study is to estimate the risk of being diagnosed with cancer in the population (excluding multiple primary cancers), while the lifetime risk of dying from cancer is to estimate the risk of dying from cancer in the population. Commonly used algorithms include cumulative rate, cumulative risk, and life table method. The AMP method used in this study is based on the life table method and adjusts for the proportion of multiple primary cancers in the population.

Here we give a briefly introduction about the methods of lifetime risk of developing or dying from cancer as follows:

1. Cumulative rate

A denotes the upper limit of age-band, usually use 74 years for cumulate rate calculation. The incidence/mortality rate is usually calculated by five-year age groups, i.e., 0-4, 5-9, …, 80-84, 85+ years.

W_i_ denotes the width of the i^th^ age interval

R_i_ denotes the age-specific annual incidence and mortality rate in the i^th^ age band

2. Cumulative risk

The cumulative risk can be calculated by the formular:

3. The “adjusted for multiple primaries (AMP)” method

In an ideal scenario, continuous follow-up of a cohort for an entire lifetime is essential to determine precise lifetime risk of developing or dying from cancer. In reality, however, it's very difficult to follow a cohort throughout their lives, so estimates based on current data require reasonable assumptions. The AMP method in this study is based on some assumptions: (1) The risk of developing a new primary cancer is equivalent between individuals without prior cancer diagnosis and the general population. (2) Mortality rates for non-cancer causes are comparable between those without previous cancer and the general population. (3) the cancer incidence, mortality, and the all-cause mortality rates at different ages in the year 2020 can be applied to a cohort as people age, which means the current age-specific rates will be maintained for a long time.

The AMP method has been introduced in detail in previously study (**PD Sasieni et al, Br J Cancer (2011) 105, 460 – 465; Ahmad AS,** **Br J Cancer (2015);112:943-7**), and here is a brief introduction.

Let denotes the cancer incidence rate at age a, and let denotes the probability of being alive and cancer-free at age a, then mathematical, the lifetime risk of getting cancer can be calculated as

But and cannot be directly estimated from routinely registry data, and in practice, the real data is usually presented as discrete data for a 5-year age bands (e.g. 0-4, 5-9, …, 80-84, 85+ years), therefore, we can estimate the lifetime risk as follows:

where for age group i:

 denotes the probability of being diagnosed with cancer

M_i_ denotes the annual number of deaths (all-cause mortality)

D_i_ denotes the annual number of cancer deaths (cancer mortality)

When estimating the risk of developing cancer, R_i_ denotes the annual number of cancer cases

When estimating the risk of dying from cancer, R_i_ denotes the annual number of cancer deaths

N_i_ denotes the population

W_i_ denotes the width of the i^th^ age group, the i^th^ interval is from a_i_ to a_i+1_, w_i=_(a_i+1_-a_i_)

denotes the observed cancer incidence rate, for age group i () can be calculated as:

denotes the probability of being alive and cancer free at age i. When calculating, died individuals should be deleted to calculate the number of surviving and cancer-free individuals. In addition, individuals diagnosed with cancer should also be deleted. To avoid double counting when estimation, we must subtract the number of cancer deaths (D_i_) from all deaths (M_i_), and then add the new number of cancer cases (R_i_). So, the probability of being alive and cancer free at age i [can be approximately estimated as follows:

The cancer incidence and mortality data are presented in the form of frequency table for calculation, and the final age band is usually 85 years old or above (e.g. 0-4,5-4,10-10, …, 80-84, 85+). For the final age band (e.g. 85+), the integral for the final age band evaluates to the following:

**The derivation of variance to the following:**

let：

then

=

Let:

 then

, let then

According to delta method, if, then. Let

,

Let
, and，then

Var(

Let according to delta method，then：

(7)

 95% confidence intervals=±1.96

**Reference:**

1. Sasieni P, Shelton J, Ormiston-Smith N, Thomson CS, Silcocks PB. What is the lifetime risk of developing cancer?: The effect of adjusting for multiple primaries. Brit J Cancer 2011; 105:460-5

2.Ahmad AS, Ormiston-Smith N, Sasieni PD. Trends in the lifetime risk of developing cancer in Great Britain: comparison of risk for those born from 1930 to 1960. Br J Cancer 2015; 112:943-7.
